# Immunoinformatics Based Study of T Cell Epitopes in Zea m 1 Pollen Allergen

**DOI:** 10.3390/medicina55060236

**Published:** 2019-06-01

**Authors:** Anamika Basu

**Affiliations:** Deptartment of Biochemistry, Gurudas College, Kolkata 70006, India; basuanamikaami@gmail.com

**Keywords:** peptide-based vaccine design, Zea m 1, pollen allergens, type I hypersensitivity reaction, molecular docking

## Abstract

*Background and Objectives:* Zea m 1 is a pollen allergen, which is present in maize, is accountable for a type I hypersensitivity reaction in all over the world. Several effective medications are available for the disorder with various side effects. Design and verification of a peptide-based vaccine is a state-of-art technology which is more cost effective than conventional drugs. *Materials and Methods:* Using immunoinformatic methods, the T cell epitopes from the whole structure of this allergenic protein can be predicted. Worldwide conserved region study among the other pollen allergens has been performed for T cell predicted epitopes by using a conservancy tool. This analysis will help to identify completely conserved HLA (human leukocyte antigen) binding epitopes. Lastly, molecular docking study and MHC-oligopeptide complex binding energy calculation data are applied to determine the interacting amino acids and the affinity of the epitopes to the class II MHCmolecule. *Results:* The study of criteria-based analysis predicts the presence of two epitopes YVADDGDIV and WRMDTAKAL on this pollen allergen. *Conclusions:* The T cell epitopes identified in this study provide insight into a peptide-based vaccine for a type I hypersensitivity reaction induced by Zea m 1 grass pollen allergenic protein.

## 1. Introduction

An epitope is a portion of an antigenic protein molecule that can be identified by the immune system of the human body by either B or T lymphocyte cells. The peptides can be interacted with through the T-cell receptors of the immune system. After being bound to at least one Major Histocompatibility Complex (MHC) protein, the antigenic proteins are intracellularly processed and exposed on the surface of the antigen presenting cell, known as the MHC-peptide complex. Since T lymphocytes play an active role in this type of immune response along with the specific MHC molecule from the antigen presenting cell for antigen presentation, this type of epitopes is therefore called a T-cell epitope [1]. MHC molecules are cell surface glycoproteins, which actively contribute to host immune reactions. These MHC molecules are expressed from human HLA (Human Leukocyte Antigens) gene to participate in adaptive immune system in humans [2].

The immunogenicity of T cell epitope is dependent on various factors e.g., suitable and actual peptide processing from its protein source, stable small peptide binding with the MHC molecule and lastly, recognition of the MHC-bound peptide molecule by the T cell receptor [3]. Two types of MHC molecules known as MHC class I molecules normally present peptides containing 8 to 11 amino acids in length, but the peptides binding to MHC class II may have peptide lengths that vary from 12 to 25 amino acids [4]. MHC class II protein molecules bind with fragments of the oligopeptide, obtained from the proteolytic cleavage of antigenic protein and present them on the cell surface of antigen presenting cells (APCs), to be recognized by CD4+ T cells. When adequate amounts of the epitope are presented, the T cell may generate a specific adaptive immune response for that pathogen through the process of positive selection. Class II MHC molecules are secreted on specialized cells, e.g., professional APCs such as B cells, macrophages and dendritic cells, whereas class I MHCs are expressed on every nucleated cell of the human body [5].

In an ideal peptide-based vaccine, both B- and T-cell epitopes are present [2]. B cell and T cell epitopes are recognized by two different pathways. The three-dimensional conformation of the antigenic protein molecule is wholly responsible for recognition by B cells, whereas T cells can recognize an antigen protein molecule only after it has been digested to form a small peptide fragment which must be bound with the major histocompatibility complex (MHC) class II molecule, forming a ternary complex.

Allergic symptoms are one of the most common health problems in the world. Among the four types of hypersensitivity reactions, more than 25% of the world’s population suffers from a type I hypersensitivity reaction. Considering the various causes of allergic reactions, pollen allergens are predicted to be the most potential source of hypersensitivity reaction. Pollinosis due to various pollens results in allergic rhinitis and asthma [6]. Grass pollen allergens from *Cynedondactylon*, *Orzya sativa*, *Zea mays* etc. are accountable for the allergic reaction in susceptible individuals in different parts of the world [7]. Allergic diseases can be successfully treated by identifying clinically important allergens. Worldwide, about 400 million people are suffering from hay fever as well as seasonal asthma [8]. The major causative biomolecules for this allergy are pollen proteins also known as the group-1 grass pollen allergens [9]. Zea m 1 is found in a class of abundant grass pollen allergens, which are formed by several genes. These proteins can loosen the walls of grass cells, including the maize stigma and style [10]. In a study, performed in Portugal on thirty-two children that are under 8 years of age, all are given a positive skin-prick test for grass pollen allergens [11]. 38% of the children are monosensitized to different grass pollen allergens. The decreasing order of sensitization frequency for that pollens are shown in Table 1.

To prevent the type I hypersensitivity reaction, drugs known as mast cell stabilizers e.g., synthetic, semi synthetic and natural stabilizers, can be used as therapeutic agents. These mast cell stabilizers obtained from natural resources can be classified into different groups such as flavonoids, coumarins, phenols [12], terpenoids, alkaloids etc. Considering their mechanisms of action, mast cell stabilizers can be classified as Ca^2+^channel blocking agents, suppressors of gene expression of genes (tumor necrosis factor alpha, different interleukins), inhibitors for phosphorylation reactions in MAPK, ERK, JNK Gab2 signaling pathways, down regulators of enzyme histidine dicarboxylase, suppressors of mRNA of CD23, inhibitors of COX2/5 lipooxygenase enzyme, inhibitors of prostaglandin D_2_ synthesis, LTC_4_ inhibitors, and Spleen tyrosin kinase enzyme inhibitors [13]. Explorations for alternative types of therapeutics in allergic reactions are explained in my earlier work where, a specific sense siRNA is explored as anti-allergic therapeutic during an immediate type of hypersensitivity reaction, caused by the Zea m 1 pollen allergenic protein [14]. Presently, in allergen-specific immunotherapy (AIT), the disease-causing allergens are used for a disease-modifying treatment of allergy. The molecular allergen characterization process is applied, to produce allergy vaccines with the recombinant allergens, peptides and genes synthesizing allergens. The B-cell epitope technique is also another promising method used to identify the antigenic determinants or epitopes present in the antigenic proteins [15]. Epitope based vaccine design is a state-of-art method, because it is very specific, is able to evade undesirable immune reactions, has the power to create long lasting immunity, and at the same time, it is cheap in price. This method has been applied to treat various diseases like tuberculosis [1], Nipah virus infection [16] etc. In this study, an epitope-based peptide vaccine design method is studied for Zea m 1 pollen allergen, with various T cell epitope prediction methods, followed by molecular docking technique. Prediction methods and docking experiments are performed to design peptide-based vaccines for an allergic reaction caused by Zea m 1 pollen allergen.

## 2. Materials and Methods

**Figure A1:**
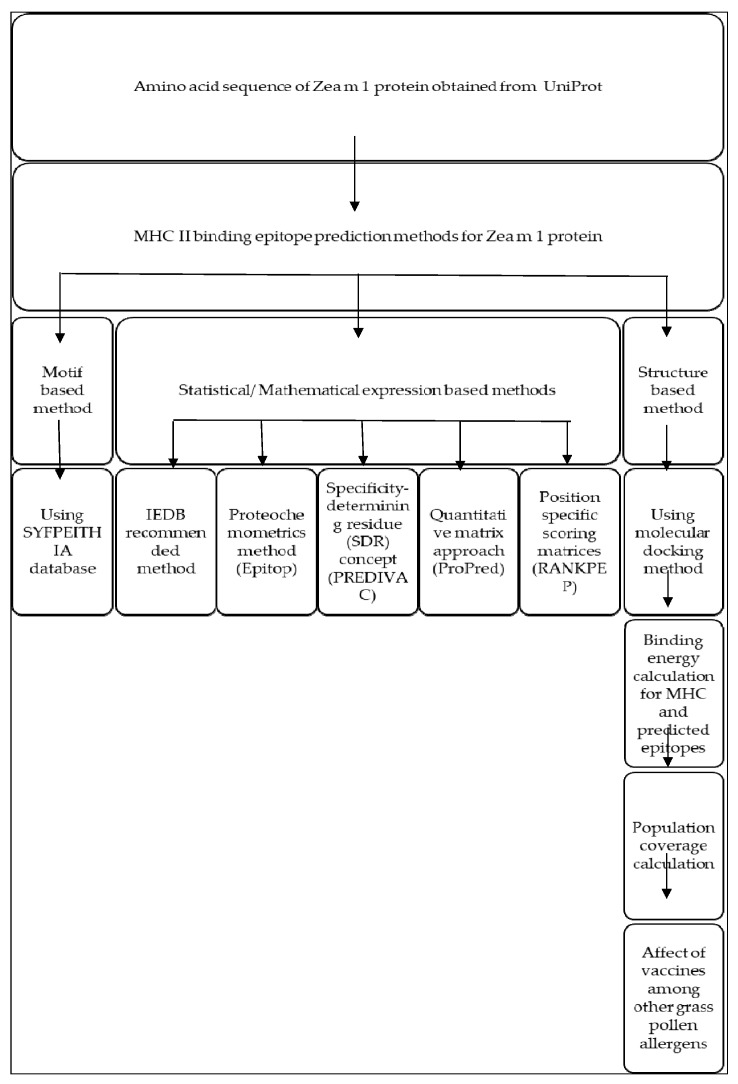
Schematic representation for T cell epitope prediction in Zea m 1.

Different steps and computational methods applied to forecast T cell epitopes for preparing peptide-based vaccine for Zea m 1 pollen allergen areshown in Figure A1.

### 2.1. Retrieval of Zea m1 Pollen Allergen Protein in FASTA Format

The amino acid sequence and three-dimensional structure of Zea m 1 allergenic protein (PDB ID 2HCZ) are obtained from UniProt knowledgebase [17].

### 2.2. MHC II Binding Epitope Prediction for Allergenic Protein

The MHC binding epitope prediction methods for Zea m 1 allergen can be classified into three groups such as (i) methods based on protein motifs, (ii) expression-based methods using statistics or mathematics and, (iii) methods based on structure of the allergen.

#### 2.2.1. Motif Based Methods

SYFPEITHIA DATABASE OF MHC LIGANDS AND PEPTIDE MOTIFS (Ver. 1.0) (http://www.syfpeithi.de/), is a database which comprises more than 7000 peptide sequences, known to bind class I and class II MHC molecules. Using FASTA sequence of Zea m 1 pollen allergen, epitopes for MHC II binding are searched.

#### 2.2.2. Statistical/Mathematical Expression-Based Methods

##### IEDB Recommended Method

The IEDB recommended method (www.iedb.org) is used to identify a T cell epitope, in which the Consensus approach, in combination with NN-align, SMM-align, CombLib and Sturniolo algorithms are applied. Here a NetMHCIIpan method is also used.

##### A Proteochemometrics Based Method

EpiTOP, a proteochemometrics based model theoretically predicts peptide binding to a whole group of MHC proteins (http://www.pharmfac.net/EpiTOP). This method helps to detect T cell epitopes on the basis of mathematical expression.

##### Specificity-Determining Residue (SDR) Concept

PREDIVAC, a method based [18] on the specificity-determining residue (SDR) concept which covers 95% of MHC class II allelic variants. SDRs consist of a trivial set of structurally conserved locations in the peptide-binding interaction interface that are responsible for specific recognition of MHC II molecules. Peptide binding prediction to the HLA class II protein DRB3*0101 is executed by parsing the query protein sequence into overlapping nonameric segments (peptides), each of which is assigned a Predivac binding score (0–100).

##### A Method is based on the QM (Quantitative Matrices) Approach

ProPred, a method is grounded on the QM (quantitative matrices) approach. It predicts binders for MHC class II molecules (http://www.imtech.res.in/raghava/propred/). This matrices-based method is also applied.

##### A MethodApplying Position Specific Scoring Matrices (PSSMs)

RANKPEP, a method (http://imed.med.ucm.es/Tools/rankpep.html) which forecasts peptide binders with the MHCII molecules from protein amino acid sequence/s or sequence alignments using Position Specific Scoring Matrices (PSSMs). Using this tool, MHC II binding epitopes of Zea m 1 are identified.

#### 2.2.3. Structure Based Prediction Method

**Figure A2:**
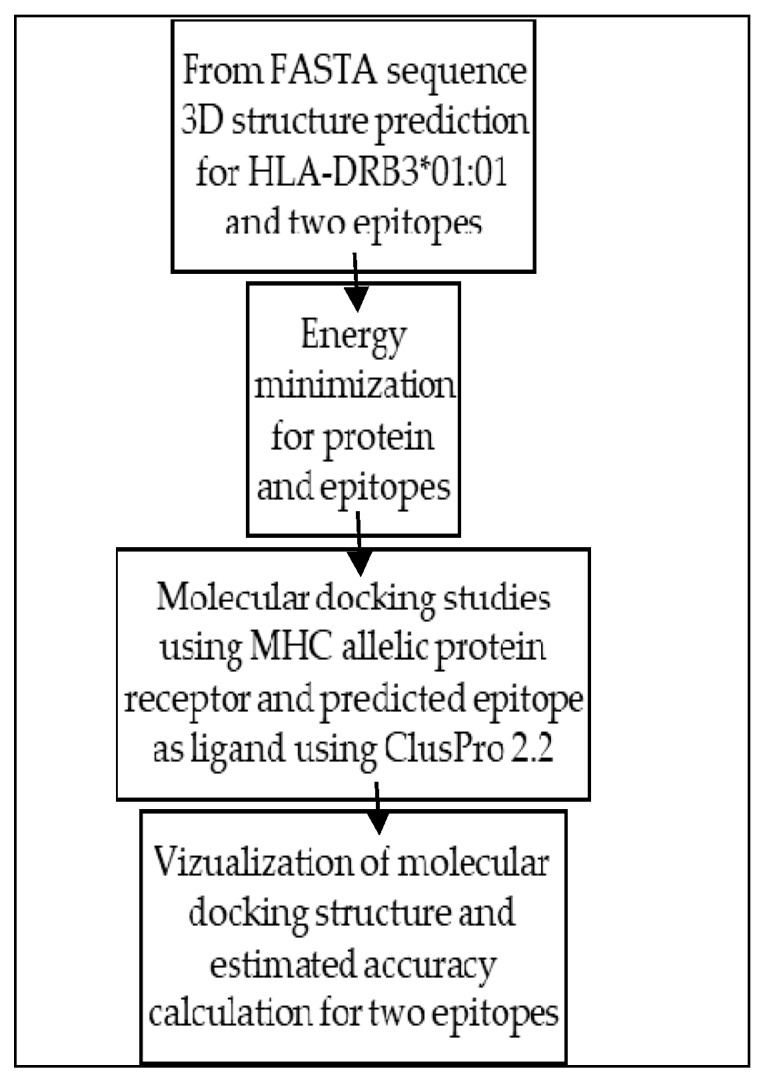
Flowchart for molecular docking procedure.

Structure based methods are based on the molecular docking technique. These methods compute binding energy between peptide and MHC molecule and the energetically favorable peptides are predicted as binders. A flowchart for molecular docking procedure is shown in Figure A2.

### 2.3. Population Coverage Prediction of Putative Epitopes

The following putative epitopes and their cumulative predicted coverage are calculated specifically for the set of HLA class II allelic variants occurring in the target population of Asia, according to allele frequency data recovered from the Allele Frequency Net Database (http://www.allelefrequencies.net/) [19].

### 2.4. Analysis for the Effectiveness of Peptide-Based Vaccine in Other Group 1 Grass Pollen Allergens

To prove the effectiveness of these two peptides as the vaccines for whole group 1 grass pollen allergens, a search is performed to identify homologous allergens in the SDAP allergens database [20]. SDAP (Structural Database of Allergenic Proteins) is a web server [20] that delivers quick access to the peptide sequences, three-dimensional structures and IgE epitopes of allergenic proteins. The database component of SDAP comprises information about the name, source, sequence, structure, IgE epitopes and literature references for allergens and easy links to the major protein from various web browsers, such as-PDB, SWISS-PROT/TrEMBL, PIR-ALN, NCBI Taxonomy Browser, as well as from literature e.g., PubMed, MEDLINE.

## 3. Results

### 3.1. Retrieved Sequence of Zea m1 Pollen Allergen Protein in FASTA Format

**Figure 1:**
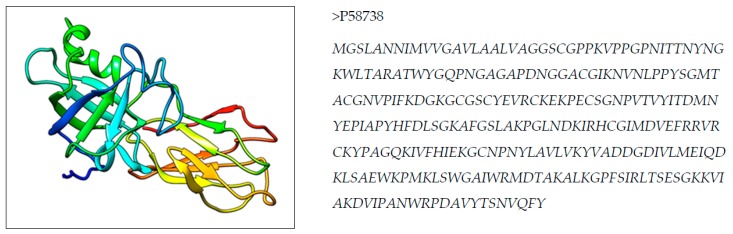
3D structure and primary sequence in FASTA of Zea m 1 pollen allergen.

An X ray crystallographic structure of Zea m 1 (PDB ID 2HCZ) is shown in Figure 1 [17]. This allergenic protein structure is used to identify a predicted T cell epitope for peptide mapping to design a vaccine.

### 3.2. T Cell Epitope Prediction for MHC II Binding

#### 3.2.1. Epitope Search Results for Motif-Based Methods

Result from SYFPEITHI, a DATABASE OF MHC LIGANDS AND PEPTIDE MOTIFS (Version 1.0), for prediction of CD4+ T cell epitope is shown in Table 2.

The peptides VKVKYVADDGDIVLM and LSWGAIWRMDTAKAL are identified as predicted T cell epitopes for this antigenic protein from SYFPEITHI database and their prediction scores for various MHC II allelic proteins are shown in Table 2.

#### 3.2.2. Predicted T Cell Epitopes by Using Statistical/Mathematical Expression-Based Methods

##### Results from IEDB Recommendation Method

The prediction method recommended by IEDB for MHC-II binding with CD4+ T cell epitope propose that the lower the percentile rank is for the epitope, the better it would be as a binder of the MHC II molecule. The predicted percentile rank using consensus and NetMHCIIpan methods for predicted T cell epitopes are shown in Table 3.

##### Results from the EpiTOP 1.0 Method

The binding affinity of predicted T cell epitopes with MHC II molecules are expressed here as IC_50_ values (Table 4).

##### Peptide Binding Prediction to the HLA Class II Protein DRB3*01:01 Using PredivacMethod

From the IEDB recommendation method, MHC II allele HLA-DRB3*01:01 is isolated as the most efficient MHC molecule for T cell binding with the lowest percentile rank (Table 3). So, this allelic protein is used for the Predivac method to predict the nanomers as T cell epitopes with predicted scores (Table 5).

##### Results for T Cell Epitope Prediction Using PROPRED Method

Predicted peptides along with their positions and predicted score in pollen allergen protein are displayed in Table 6 by using the PROPRED method.

##### Results from the RANKPEP Method

Predicted peptide sequences along with their positions and predicted score in pollen allergen protein, Zea m 1 are displayed in Table 7 by using the RANKPEP method.

#### 3.2.3. Structure Based T Cell Epitope Prediction by Using Molecular Docking Technique

**Figure 2:**
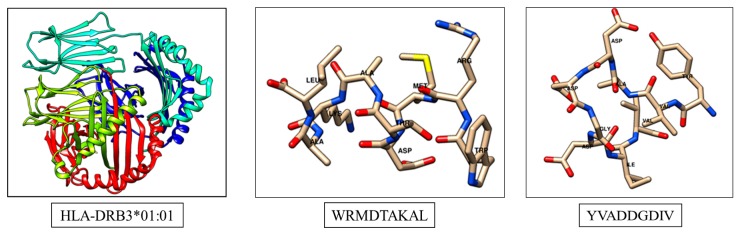
Structures of MHC molecule and predicted T cell epitopes.

From the above-mentioned methods two peptide sequences e.g. WRMDTAKAL and YVADDGDIV are selected as suitable T cell epitopes for the Zea m 1 allergenic protein. Similarly, a MHC II allele HLA-DRB3*01:01 protein is detected as the most probable interacting MHC molecule. For docking studies, both T cell epitopes are nominated and subjected to predict three-dimensional structures using a PEP-FOLD server [21,22] (Figure 2). The molecular interactions with specific HLA protein for respective epitopes are identified in docking studies with a ClusPro 2.2 web server [23].

**Figure 3:**
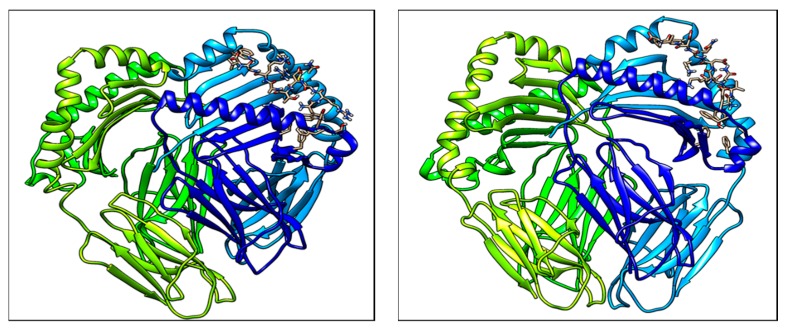
Molecular docking structures of Predicted T cell epitopes with MHC II molecule.

Molecular docking complexes for two predicted peptide structures with MHC II allele HLA-DRB3*01:01 protein are shown in Figure 3. The estimated accuracy of docked structure for peptide YVADDGDIV is higher than that of WRMDTAKAL, as shown in Table 8.

### 3.3. Population Coverage Prediction for Putative Epitopes

The following putative epitopes and their cumulative predicted coverage are calculated specifically for the cluster of HLA class II gene allelic variants present in the Asian population. Population coverage prediction is estimated considering the allelic frequency data recovered from the Allele Frequency Net Database (Table 9).

The prediction is carried out by considering 225 HLA class II proteins expressed in the target population.

### 3.4. Analysis for the Effectiveness of Peptide-Based Vaccine in Other Group 1 Grass Pollen Allergens

To prove the effectiveness of these two peptides as the vaccines for whole group 1 grass pollen allergens, a search is performed to identify homologous allergens, in the SDAP allergens database [20]. A list of allergens, using the FASTA alignments among the Zea m 1 sequence and all SDAP allergens having an E score value higher than 0.010000 are shown in Table 10.

**Figure 4:**
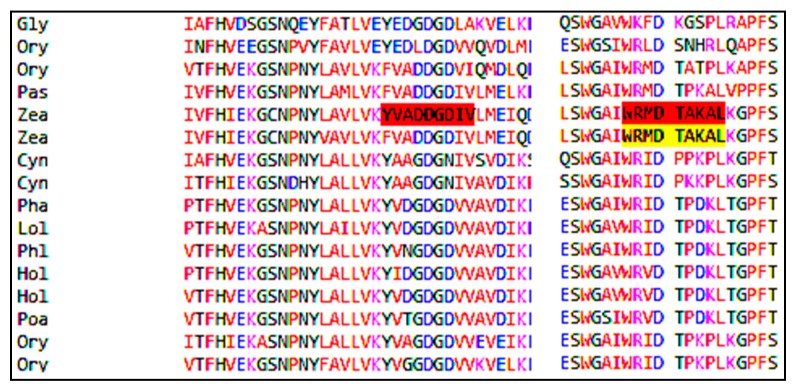
Multiple sequence alignment of two epitopes in fourteen allergens.

Among these 50 allergens, 14 grass allergens are selected, omitting isoallergenic proteins. The multiple alignment sequence study for these two T cell epitopes such as YVADDGDIV and WRMDTAKAL in 14 grass allergens, is shown in Figure 4. This study shows that the locations of amino acids present in these epitopes are almost conserved, so these two epitopes may be effective for all these 14 grass allergens after clinical verification.

## 4. Discussion

Allergens cause type I hypersensitivity reactions mediated by immunoglobulin E (IgE) molecule. IgE biosynthesis, also known as sensitization, may be caused by airborne allergens, food allergens, drug allergens and occupational allergens. Modern clinical therapeutics for allergic reactions includes a combination of patient awareness, allergenic molecule avoidance, pharmacotherapy, and allergy immunotherapy. Allergy immunotherapy is a type of treatment targeting the basic immunological molecules and immunological pathways involved in allergic reaction and resulting in the activation of immunological tolerance by reducing IgE molecule reactivity and retaining T cell molecule reactivity [24].A vast array of structurally altered allergens has been created, including allergenic oligopeptides, chemically modified allergoids, adjuvant-bound allergens, and nanoparticle-based allergy vaccines. In allergen-specific immunotherapy (AIT), repeated doses of sensitive allergens are used for desensitization or hypo-sensitization of allergic patients. Several herbal and natural products are reported to regulate antigen-IgE mediated allergic responses [25,26]. There are other types of immunotherapeutic strategies that have also reported which use idiotype and anti-idiotype antibody interaction in vaccine production [27] and epitope-paratope peptide modulation [28,29]. Different bioinformatic algorithms, as well as computational methods [30] are used for identifying biological functions of peptides.

Epitope-based vaccines are short oligopeptides derived from antigen that are used after antigen presentation to T-cells to prevent diseases like type I hypersensitivity. During antigen presentation, epitopes are bound with major histocompatibility complex (MHC) protein molecules. Peptide vaccines based on multiple T-cell epitopes can be administered for the rational use of immunogens among distinct ethnic populations, while providing numerous potential advantages over conventional vaccines. The advantages are more accurate regulation of the immune response activation, concentrating on most appropriate antigenic regions of a group of proteins (which are conserved and immunodominant in nature), as well as having advantages for production and biosafety. CD4+ T-cell epitopes display an important role in epitope-based vaccine design. The help of these cells is indispensable for the production of strong humoral and cytotoxic CD8+ T-cell responses. But the immune response to T-cell epitopes is limited only by HLA proteins. As a result, the HLA selectivity for T-cell epitopes develops a major constraint for epitope-based vaccine design, for genetically varied human populations. Two important factors cause major problems in epitope-based vaccine design. The most common problem is that MHC class II alleles are synthesized in different amounts in different ethnicities such as Asian and European populations. The second problem is that MHC class II genes are the most polymorphic genes in nature found in the human genome. Since the experimental testing of large sets of peptides of MHC molecules is very time-consuming as well as costly, in silico methods are more sought after methods to overcome these problems. Criteria-based analysis predicts two epitopes on this pollen allergen: YVADDGDIV and WRMDTAKAL. The T cell epitopes identified in this study provide insight into a peptide-based vaccine for type I hypersensitivity reaction induced by the Zea m 1 pollen allergen.

## 5. Conclusions

The crucial part of epitope-based vaccine design is its validation through in vivo and in vitro methods. Although two nonapeptides are identified by various motif based, statistical and structure-based methods, the experimental verification two epitopes YVADDGDIV and WRMDTAKAL is necessary to construct a vaccine against the Zea m 1 pollen allergen. This research work provides not only a novel pathway to design a peptide-based vaccine design for the Zea m1 pollen allergen, but at the same time the effectiveness of these two T cell epitopes is verified for 14 grass pollen allergens. Cross-reactivity occurs very frequently among these pollen allergen molecules due to very high sequence similarity in antigenic protein sequences. Almost conserved epitope sequences in these homologous proteins indicate the effectiveness of these predicted epitopes as probable vaccines for group 1 grass allergens. Population coverage calculation shows their efficiency in Asian populations. From a diagnostic point of view, these two T cell epitopes have immense importance for detecting the sensitivity of an individual towards the Zea m 1 pollen allergen. By preparing a monoclonal antibody with these two epitopes, diagnosis of a susceptible individual for hypersensitivity reaction in contact with a grass pollen allergen is possible. Allergen immune therapy with YVADDGDIV and WRMDTAKAL epitopes, will reduce immunogenic reactions in Zea m 1 sensitive populations.

## Figures and Tables

**Table 1:** Sensitization frequency of different grass pollen allergens

No.	Sources of Grass Pollen Allergens	Sensitization Frequency
1.	*Dactylis*	94%
2.	*Hordeum*	75%
3.	*Phleum*	72%
4.	*Poa*	69%
5.	*Avena*	66%
6.	*Festuca*	63%
7.	*Triticum*	59%
8.	*Secale*	53%
9.	*Lolium*	50%
10.	*Maize*	31%

**Table 2:** Predicted epitopes from SYFPEITHI database

Interacting MHC II Alleles	Predicted T Cell Epitopes	Prediction Score
HLA-DRB1*0101 15—mers	V K V K Y V A D D G D I V L M	8
HLA-DRB1*0301 (DR17) 15—mers	V K V K Y V A D D G D I V L M	0
HLA-DRB1*0401 (DR4Dw4) 15—mers	V K V K Y V A D D G D I V L M	12
HLA-DRB1*0701 15—mers	V K V K Y V A D D G D I V L M	20
HLA-DRB1*1101 15—mers	V K V K Y V A D D G D I V L M	1
HLA-DRB1*1501 (DR2b) 15—mers	V K V K Y V A D D G D I V L M	4
HLA-DRB1*0101 15—mers	L S W G A I W R M D T A K A L	11
HLA-DRB1*0301 (DR17) 15—mers	L S W G A I W R M D T A K A L	0
HLA-DRB1*0401 (DR4Dw4) 15—mers	L S W G A I W R M D T A K A L	6
HLA-DRB1*0701 15—mers	L S W G A I W R M D T A K A L	4
HLA-DRB1*1101 15—mers	L S W G A I W R M D T A K A L	6
HLA-DRB1*1501 (DR2b) 15—mers	L S W G A I W R M D T A K A L	14

**Table 3:** T cell prediction results from IEDB recommendation method.

Allele	Start	End	Peptide	Method Used	Percentile Rank
HLA-DRB3*01:01	216	230	LSWGAIWRMDTAKAL	Consensus (comb.lib./smm/nn)	0.01
HLA-DRB3*01:01	217	231	SWGAIWRMDTAKALK	Consensus	0.01
HLA-DRB3*01:01	218	232	WGAIWRMDTAKALKG	Consensus	0.01
HLA-DRB3*01:01	219	233	GAIWRMDTAKALKGP	Consensus	0.01
HLA-DRB3*01:01	220	234	AIWRMDTAKALKGPF	Consensus	0.01
HLA-DRB3*01:01	221	235	IWRMDTAKALKGPFS	Consensus	0.01
HLA-DRB3*01:01	187	201	VLVKYVADDGDIVLM	Consensus	0.12
HLA-DRB3*01:01	188	202	LVKYVADDGDIVLME	Consensus	0.12
HLA-DRB4*01:01	194	208	DDGDIVLMEIQDKLS	Consensus	0.14
HLA-DRB4*01:01	195	209	DGDIVLMEIQDKLSA	Consensus	0.14
HLA-DRB4*01:01	196	210	GDIVLMEIQDKLSAE	Consensus	0.14
HLA-DRB3*01:01	186	200	AVLVKYVADDGDIVL	Consensus	0.15
HLA-DRB4*01:01	193	207	ADDGDIVLMEIQDKL	Consensus	0.15
HLA-DRB3*02:02	218	232	WGAIWRMDTAKALKG	NetMHCIIpan	0.15
HLA-DRB1*08:02	218	232	WGAIWRMDTAKALKG	Consensus (smm/nn/sturniolo)	0.17
HLA-DRB4*01:01	192	206	VADDGDIVLMEIQDK	Consensus	0.18

**Table 4:** Predicted T cell epitopes from EpiTOP 1.0 tool.

Position	Peptide	log (1/IC50)
189	VKYVADDGD	5.740
188	LVKYVADDG	5.740
187	VLVKYVADD	5.740
185	LAVLVKYVA	5.740
191	YVADDGDIV	5.740
192	VADDGDIVL	5.740
200	LMEIQDKLS	5.740
199	VLMEIQDKL	5.740
198	IVLMEIQDK	5.740
184	YLAVLVKYV	5.740
176	IEKGCNPNY	5.740
159	FRRVRCKYP	5.740
157	VEFRRVRCK	5.740
155	MDVEFRRVR	5.740

**Table 5:** Predicted epitopes using the Predivac method

ID	Nanomer	Score	Start	End
1	WRMDTAKAL	88.29	222	231
2	VADDGDIVL	85.73	192	201
3	YVADDGDIV	77.06	191	200
4	YHFDLSGKA	73.82	128	137
5	IAKDVIPAN	71.61	247	256
6	MDTAKALKG	70.21	224	233
7	IFKDGKGCG	67.41	87	96

**Table 6:** Predicted T cell epitopes by the PROPRED method

Rank	Sequence	At Position	Score	% of Highest Score
1	WRPDAVYTS	255	6.6500	73.08
2	WRMDTAKAL	221	4.0000	43.96
3	YHFDLSGKA	127	3.9000	42.86
4	MVVGAVLAA	8	3.5000	38.46
5	IFKDGKGCG	86	2.5000	27.47
6	IRHCGIMDV	148	2.5000	27.47
7	YVADDGDIV	190	2.4000	26.37
8	IVLMEIQDK	197	2.4000	26.37
9	VRCKEKPEC	99	2.2000	24.18
10	MEIQDKLSA	200	2.1000	23.08
11	WGAIWRMDT	217	1.9000	20.88

**Table 7:** Predicted results from the RANKPEP method.

RANK	POS.	N	SEQUENCE	C	MW (Da)	SCORE	% OPT.
1	222	GAI	WRMDTAKAL	KGP	1050.27	18.994	39.37%
2	221	WGA	IWRMDTAKA	LKG	1050.27	17.006	35.25%
3	50	RAT	WYGQPNGAG	APD	907.97	14.588	30.24%
4	211	SAE	WKPMKLSWG	AIW	1068.36	12.582	26.08%
5	256	PAN	WRPDAVYTS	NVQ	1053.18	12.238	25.37%
6	15	GAV	LAALVAGGS	CGP	739.87	11.915	24.70%
7	76	LPP	YSGMTACGN	VPI	884.97	11.738	24.33%
8	191	LVK	YVADDGDIV	LME	948.0	11.246	23.31%
9	122	DMN	YEPIAPYHF	DLS	1118.28	8.83	18.30%
10	43	NGK	WLTARATWY	GQP	1103.31	8.514	17.65%

**Table 8:** Docking results for two predicted epitopes.

Model	Protein Template	Peptide Template	Protein Structure Similarity (TM-Score)	Interaction Similarity Score	Estimated Accuracy
1	2Q6W_B	2Q6W_C	1.000	177.0	1.000
2	2Q6W_B	2Q6W_C	1.000	124.0	0.904

**Table 9:** Predicted population coverage for predicted peptides.

Peptide	Cumulative Coverage	Protein Localization	
	(%)	Start	End
IMVVGAVLA	60.17	8	16
MTACGNVPI	74.55	79	87
IPANWRPDA	91.42	252	260
FGSLAKPGL	91.49	137	145
YVADDGDIV	96.13	191	199
MGSLANNIM	96.16	1	9
IWRMDTAKA	96.40	221	229
YHFDLSGKA	96.86	128	136
FHIEKGCNP	96.87	174	182

**Table 10:** List of homologous allergens of Zea m1 in the SDAP allergen database.

No	Allergen	Sequence Link in SwissProt/NCBI/PIR	Sequence Length	Bit Score	E Score
1	Zea m 1	P58738	269	342.2	9.0 × 10^−96^
2	Pas n 1.0101	ACA23876	265	294.2	2.4 × 10^−81^
3	Ory s 1	AAF72990	269	270.3	4.0 × 10^−74^
4	Zea m 1	Q07154	191	261.1	1.6 × 10^−71^
5	Ory s 1	AAF72983	267	237.9	2.2 × 10^−64^
6	Ory s 1	AAF72991	267	235.8	9.3 × 10^−64^
7	Phl p 1	P43213	263	223.7	4.3 × 10^−60^
8	Phl p 1.0101	CAA81613	263	223.3	5.6 × 10^−60^
9	Cyn d 1	O04701	246	221.7	1.6 × 10^−59^
10	Hol l 1.0102	CAA93121	248	220.5	3.5 × 10^−59^
11	Hol l 1	P43216	265	220.4	4.0 × 10^−59^
12	Hol l 1	CAA10140	263	218.9	1.2 × 10^−58^
13	Cyn d 1	AAL14078	262	217.4	3.3 × 10^−58^
14	Cyn d 1.0203	AAL14079	262	216.8	4.9 × 10^−58^
15	Cyn d 1.0202	AAL14077	262	216.8	4.9 × 10^−58^
16	Poa p a	CAA10520	263	216.4	6.4 × 10^−58^
17	Pha a 1	Q41260	269	216.3	7.4 × 10^−58^
18	Cyn d 1.0204	AAF80379	244	215.8	9.4 × 10^−58^
19	Cyn d 1.0201	AAK96255	244	214.8	1.8 × 10^−57^
20	Lol p 1.0103	CAB63699	263	214.9	1.8 × 10^−57^
21	Ory s 1	AAF72987	275	214.8	2.1 × 10^−57^
22	Lol p 1.0102	AAA63278	252	213.9	3.5 × 10^−57^
23	Lol p 1.0101	AAA63279	263	213.2	6.0 × 10^−57^
24	Lol p 1	P14946	263	212.8	7.9 × 10^−57^
25	Ory s 1	AAF72984	268	210.7	3.4 × 10^−56^
26	Ory s 1	AAF72985	286	207.8	2.8 × 10^−55^
27	Tri a ps93	AAD10496	271	205.0	1.8 × 10^−54^
28	Ory s 1	AAB61710	261	204.8	2.0 × 10^−54^
29	Ory s 1	Q40638	263	200.6	3.6 × 10^−53^
30	Sor h 1.0101	ABC58726	239	199.6	6.9 × 10^−53^
31	Ory s 1	AAF72989	271	197.4	3.4 × 10^−52^
32	Ory s 1	AAF72988	327	165.7	1.5 × 10^−42^
33	Gly m 2	AAA50175	277	154.8	2.3 × 10^−39^
34	Ory s 1	AAF72986	275	144.9	2.2 × 10^−36^
35	Arat expansin	CAB37496	265	90.5	5.3 × 10^−20^
36	Ory s 1	AAG13596	275	83.7	6.1 × 10^−18^
37	Ory s 1	BAA85432	284	81.4	3.0 × 10^−17^
38	Cyn d 2	CAA10346	122	53.4	3.5 × 10^−9^
39	Dac g 2	CAA10345	122	52.7	5.9 × 10^−9^
40	Poa p 2	CAA10348	122	52.7	5.9 × 10^−9^
41	Phl p 2	P43214	122	52.7	5.9 × 10^−9^
42	Lol p 2	P14947	97	48.8	7.0 × 10^−8^
43	Dac g 3	P93124	96	48.0	1.2 × 10^−7^
44	Lol p 2	CAA51775	88	46.4	3.3 × 10^−7^
45	Phl p 3.0101	2JNZ_A	108	44.5	1.5 × 10^−6^
46	Dac g 1.0101	Q7M1X8	34	42.4	2.0 × 10^−6^
47	Lol p 3	P14948	97	43.1	3.6 × 10^−6^
48	Cyn d 15	AAP80171	112	43.2	3.7 × 10^−6^
49	Ant o 1.0101	Q7M1X6	32	38.9	2.2 × 10^−5^
50	Tri a 3	CAA90746	118	39.7	4.6 × 10^−5^
